# Research on Compressive Strength of Manufactured Sand Concrete Based on Response Surface Methodology

**DOI:** 10.3390/ma17010195

**Published:** 2023-12-29

**Authors:** Kang Gao, Zhenjiao Sun, Hui Ma, Guanguo Ma

**Affiliations:** 1 College of Safety and Environmental Engineering, Shandong University of Science and Technology, Qingdao 266590, China; skdgk@sdust.edu.cn (K.G.); sunzhenjiao@foxmail.com (Z.S.); 2 School of Safety and Engineering, China University of Mining and Technology, Xuzhou 221116, China; hughie_ma@163.com

**Keywords:** siliceous sand concrete, composite admixtures, response surface method (RSM), compressive strength

## Abstract

Due to the impact of economic and social development on the environment, there is an increasing demand for manufactured sand to replace natural sand as fine aggregate for concrete. At the same time, the effect of admixtures on the rheological properties and compressive strength of concrete is crucial in civil engineering applications. In this study, with the Box–Behnken test model, we analyzed and investigated the impact of a composite admixture of stone powder (SP), pulverized fuel ash (PFA), and silicon fume (SF) on the compressive strength of siliceous manufactured sand concrete using response surface methodology (RSM). At the same time, the rheological properties of the siliceous artificial sand and river sand concrete were analyzed. The prediction of the compressive strength of siliceous artificial sand concrete was developed using multiple regression analysis, the factors of which were SP, PFA, and SF content, and the response value was compressive strength. Furthermore, response surface and contour lines were used to analyze the impact of composite admixtures. It is shown that the compounding of SP, PFA, and SF improve the rheological properties of manufactured sand concrete. For the single factor, SP has the greatest effect on the compressive strength of mechanism sand concrete and SF has the least effect. For compounding, SP and PFA have the most significant effect on the compressive strength of artificial sand shotcrete, and the compounding of PFA and SF have the least effect.

## 1. Introduction

Since the invention of concrete, natural river sand has been an important aggregate in concrete. However, with rapid economic and social development, river sand is no longer sufficient to meet the needs of construction projects. Moreover, excessive extraction of natural river sand has severely impacted the natural environment. Therefore, scholars in various countries have begun to study the use of manufactured sand instead of river sand to formulate concrete [1,2,3,4,5]. According to relevant statistical data, in the last decade, the proportion of artificial sand used in various engineering construction fields in China has been increasing year by year. Now, manufactured sand accounts for over 80% of the total consumption of sand and stone aggregates [6,7,8].

Manufactured sand, produced through processes like crushing, screening, and shaping of ore, differs significantly in composition from natural sand. Current research indicates that the performance of concrete made with mechanical sand differs from that of natural sand concrete. In some aspects, concrete made with manufactured sand even outperforms that made with natural sand. However, it may also exhibit inferior properties such as brittleness, which is closely related to rock lithology and crushing processes [9,10,11,12].

The addition of various mineral admixtures has effectively improved the workability of concrete made with manufactured sand [13,14,15]. The presence of a certain amount of SP is a significant distinction between manufactured sand and natural sand. Within a specific range, the SP can fill voids, optimize grading, enhance concrete workability, induce crystallization of hydration products, promote cement hydration, and effectively enhance the mechanical properties and resistance to chloride ion penetration of concrete made with manufactured sand [16,17,18]. PFA can facilitate the flocculation process in cement hydration, reducing water requirement and filling the porosity to hinder agglomeration between cement particles. The incorporation of SF into concrete significantly improves the cohesion and adhesion of shotcrete, increasing the thickness of each layer. Moreover, studies have shown that adding SP, PFA, and SF to concrete made with artificial sand can have a “cumulative effect”, reducing the heat of hydration and enhancing mechanical properties [19,20,21].

Ding X et al. [22] analyzed the effect of SP level on the compressive strength of concrete at 358 days, indicating that an SP content below 13% is advantageous for the later-stage compressive strength. Wang Q et al. [23] suggested that for medium- to low-strength pumped concrete, the optimal SP content ranges from 22% to 25%. Knop Y et al. [24,25] demonstrated through experiments that substituting an appropriate amount of SP for cementitious materials can increase the filling density of the cement system, thereby improving the performance of the cement slurry and enhancing the compressive strength of concrete. Ding H et al. [26] investigated the effect of single additions of PFA, single additions of mineral powder, and combined additions of PFA and mineral powder on the performance and strength of concrete made with mechanism sand.

Peng Y et al. [27] examined the influence of lime SP content on flowability, setting time, flexural and compressive strength, shrinkage, and permeability of cement mortar. When the content of lime SP increased from 5% to 28%, the setting time of the mortar continuously shortened, and the flowability, flexural and compressive strength, and 120-day contractions showed an increase followed by a decrease, while the permeability was minimally affected by the lime SP content. Fu T et al. [28] used a three-phase composite model to predict the elastic modulus of SP concrete. The slump, expansion, compressive, and splitting strengths of concrete made with manufactured sand increased initially and then decreased with an increase in SP content. The slump and expansion were maximal at an SP content of 9%, while the compressive and splitting strengths at 7 and 28 days were maximal at an SP content of 12%. Currently, most research is based on the single addition of SP, PFA, or SF, or their combined addition in varying proportions, studying their effects on the rheological and mechanical properties and hydration of concrete made with mechanism sand. However, research on the comprehensive effects of the three under the influence of mechanism sand concrete is lacking.

Response surface methodology uses graphical techniques to display the response of a system as a functional relationship of one or more factors. Thus, we can determine the optimization conditions in the experimental design by visual observation. The Box–Behnken design in RSM has been widely used in engineering applications since its proposal [29]. Research on additive dosage and concrete using this design has been a constant focus. Rajesh and Kumar [30] optimized concrete with good hardening and functional performance using the Box–Behnken design of RSM. Khudhair M H et al. [31], using the RSM of experimental design, determined a predictive function model for the compressive strength of high-capacity concrete prepared with a high-efficiency water reducer and a setting accelerator (SP103) based on component proportions.

In summary, the effect of the combined use of various mineral admixtures on the performance of concrete made with manufactured sand requires further research. RSM can be fitted to obtain a relationship between factors and response values. Based on research using RSM Box–Behnken, this research uses natural sand and silica-based manufactured sand as basic raw materials, considers the content of SP, PFA, and SF as factors, and compressive strength is taken as the response value to investigate the influence of the three additives on the compressive strength of concrete made with mechanism sand. Furthermore, the rheological properties of concrete made with mechanism sand and natural sand are analyzed to explore the effect mechanisms of combined mineral admixtures on the performance of concrete made with manufactured sand. This study aims to establish a multivariate predictive regression model for various influencing factors using response surface methodology (RSM). The goal is to provide experimental evidence and theoretical guidance for the mix design of silica-rich engineered sand concrete.

## 2. Experimental Preparation

### 2.1. Experimental Materials

Cement: The common portland cement PO42.5 produced by Shandong Shanshui Cement Group (Rizhao, China) Co., Ltd., and was used for the experiments. Its quality was in accordance with the GB175-2020 and ASTM C150 [32,33]. The composition is shown in Table 1. The cement used in the experiment had a specific surfactant area of 338 m^2^/kg and a loss on ignition of 4.54%. The initial setting time of the cement was greater than 45 min. The final setting time was less than 600 min.

Water: All the water used in the experiment came from tap water and met the requirements of the JGJ63-2006 [34].

**Figure 1:**
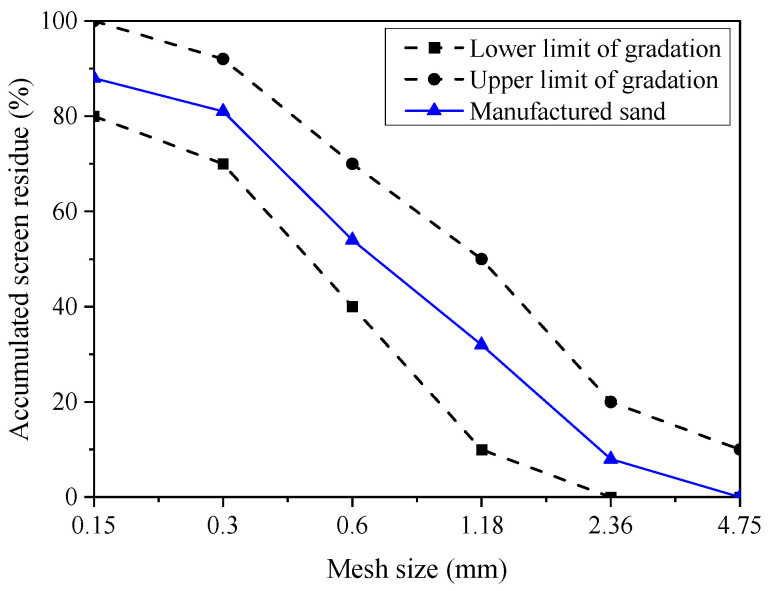
Aggregate grading diagram.

Manufactured sand and coarse aggregates: All the artificial sand in this study was taken from the Phase 1 project of Qingdao Metro Line 6 in Shandong Province and was classified as silica sand. The construction of Qingdao Metro involved blasting, resulting in large chunks that needed to be crushed and screened before use. In this study, a jaw crusher was employed to crush stones. The engineered sand gradation was utilized as depicted in Figure 1. Fine aggregates were sieved using standard sieves with nominal diameters of 0.16 mm, 0.315 mm, 0.63 mm, 1.25 mm, 2.50 mm, and 5.00 mm, and aggregates within the particle size range of 0.16 mm to 5 mm were selected. Coarse aggregates were sieved using standard sieves with nominal diameters of 5 mm and 10 mm, and aggregates within the particle size range of 5 mm to 10 mm were chosen. The aggregate gradation complied with the GB50086-2015 [35]. Coarse aggregates consisted of durable crushed stone with a particle size of 5–10 mm complied with the GB50086-2015.

Stone powder (SP): SP content can be good for concrete workability within a certain range. However, exceeding this range can have negative effects. Excessive SP with high water-cement ratio concrete can help lower the water-cement ratio, enhance cohesiveness and water retention, and reduce bleeding. In cases with good workability, excessive SP content can result in a lower water-cement ratio, decreasing the flowability of the mix [16,17,27]. In this study, SP was obtained by screening and crushing manufactured sand, with a particle size <0.075 mm. The dosage of SP was set at three levels—5%, 10%, and 15%—of the cement weight.

Pulverized fuel ash (PFA): PFA has reactive and filling properties that increase the structural density of concrete. In the early stages of concrete mixing and formation, the morphology effect and macroaggregate filling effect of PFA can ameliorate the workability of concrete [26,36]. For shotcrete, better pumpability and application can be achieved. The PFA used in this study was F-class PFA produced by Henan Hengyuan New Materials Co., Ltd. (Xinyang, China). Its composition is shown in Table 2, meeting the requirements of Class I PFA for relevant parameters. The dosage of PFA was set at three levels—10%, 15%, and 20%—of the cement weight.

Silicon fume (SF): SF, when added in too small quantities, has little effect on concrete properties. However, excessive SF can make concrete too viscous, challenging for construction, and lead to high shrinkage and poor freeze–thaw resistance [37,38,39]. The SF used in this study was produced by Henan Hengyuan New Materials Co., Ltd. (Xinyang, China). Its composition is shown in Table 3, with a density of 2.4 g/cm^3^ and a specific surface area of 75,000 m^2^/kg. The dosage of PFA was set at three levels of 2.5%, 5.0%, and 7.5% of the cement weight.

### 2.2. Experimental Methods

#### 2.2.1. Methods of Experimental Design

Response surface methodology (RSM), also known as response surface analysis, involves designing a rational number of experiments, collecting specific data, and utilizing a multivariate quadratic regression equation to model the functional relationship between factors and response values. This method allows for the precise examination of the relationships between various factors and response values, enabling the rapid and effective determination of optimal conditions for multifactor systems. RSM offers advantages such as a reduced number of experiments, shorter experimental cycles, and high precision, making it a technique for efficiently optimizing fundamental experimental conditions.

The Box–Behnken experimental design is a commonly used method for optimizing the influence of multiple factors on a response variable. It is a type of response surface methodology that constructs experimental designs by adding a set of central points between the high and low levels of each factor. The Box–Behnken design estimates linear, quadratic, and interaction effects of factors with fewer experiments and identifies the optimal points on the curved response surface.

#### 2.2.2. Mix Proportions

The experiments were conducted using a water-cement ratio of 0.5, with the proportions of cement, crushed stone, sand, and water being 1:1.5:2.25:0.5. Based on the Box–Behnken experimental model, there were a total of 17 mix proportions for different levels of additives. Additionally, natural sand concrete without the addition of SP, PFA, and SF was used as a control group. SP (X_1_), PFA (X_2_), and SF (X_3_) are added in percentages based on their weight relative to the cement (total weight of cement and PFA). Specifically, the quantity of PFA added replaces the weight of cement, while the addition of SP and SF does not replace the weight of cement. The water-cement ratio in the mix remains constant, representing the percentage of water’s weight relative to the combined weight of cement and PFA. The composition of concrete is shown in Table 4.

#### 2.2.3. Macroscopic Characteristics and Rheological Property Testing

1. Macroscopic Characteristics

The appearance and morphology of aggregates can affect concrete’s workability, mechanical properties, durability, and resistance to erosion. In this study, macromorphological characteristics of the mechanism sand and natural sand were obtained using a Leica 3D microscope (Leica DVM5000 HD).

2. Rheological Properties

Concrete not only requires high strength and ideal durability after hardening but also demands good workability when freshly mixed. Rheological testing is essential for fresh and hardened concrete, especially when concrete needs to be pumped. Shotcrete must undergo pumping and pneumatic conveying stages, and for mining and underground engineering applications, it may require longer pumping distances. Therefore, shotcrete needs to have certain rheological properties while meeting mechanical performance requirements.

Rheological properties are tested using an eBT2 rheometer manufactured by Schleibinger. The main rheological parameters tested are relative yield shear stress and plastic viscosity coefficient. These parameters are measured using a rotating thrust probe installed on the rheometer, which records 100 angles and 100 force values per revolution.

#### 2.2.4. Specimen Preparation

**Figure 2:**
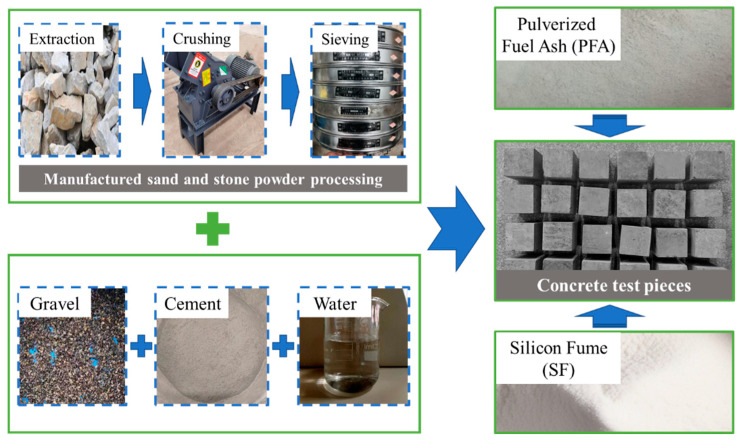
The fabrication process of the test piece.

The preparation of concrete specimens followed the standard dimensions specified in the GB/T50081-2019 [40], with mold dimensions of 100 × 100 × 100 mm. According to the concrete mix proportions and the additive ratios in Table 4, the concrete was prepared. In the experimental process, coarse aggregates, artificial sand, and cement were mixed in a concrete mixer for 1 min. Then, water and other additives were added, and mixing continued for 3 min. The mixed concrete was added to the molds and the vibrating table was placed for vibration for a period of 4 min. The final hanging off of more than concrete on the mold was cured at room temperature for 24 h and then demolded. After demolding, the specimens were cured as standard for 28 days [40]. The specimen fabrication process is illustrated in Figure 2.

#### 2.2.5. Compressive Strength

The compressive strength test adopts a DYE-2000 digital display pressure testing machine, and the test content is uniaxial compressive strength. The loading speed of the pressure tester is around 0.8 mm/min, and its autonomy records and outputs the maximum pressure value as the uniaxial compressive strength [41].

## 3. Results and Discussion

### 3.1. Macroscopic Features and Rheological Properties

1. Macroscopic Features

**Figure 3:**
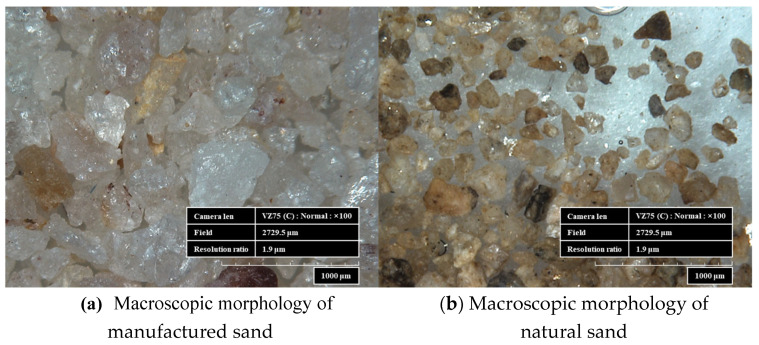
Macroscopic morphology of manufactured sand and natural sand.

The macroscopic features of the artificial sand and natural river sand used in the experiment were observed under a Leica microscope, as shown in Figure 3. The manufactured sand, produced through mechanical crushing and grinding, exhibits irregular polyhedral shapes under the influence of mechanical force. It contains some needle-like particles, with relatively few impurities, appearing clean and displaying an oily luster. The color is milky white or translucent with a shell-like fracture surface. In contrast, natural sand, formed by the prolonged impact and abrasion of natural stone by water, has smoother grains with fewer edges and corners. Its surface is relatively smooth, but it contains more impurities, displaying a complex range of colors, primarily yellow.

2. Rheological Properties

**Figure 4:**
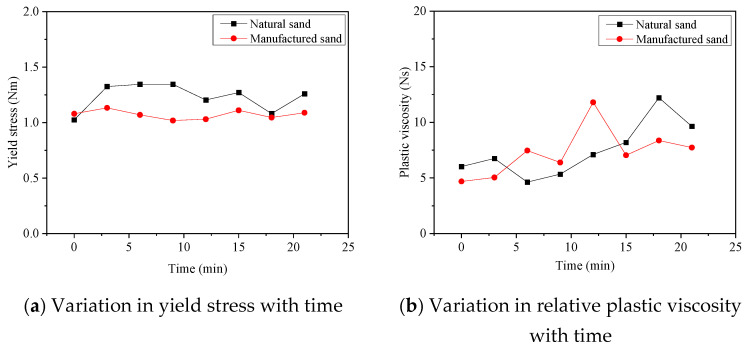
Rheological property comparison between natural sand concrete and manufactured sand concrete.

According to references [17,22,23,24,25,26,27,28], the compressive strength of mechanism sand concrete with a moderate dosage of admixtures (SP, PFA, SF at 10%, 15%, and 5%, respectively) is favorable when a single admixture is used. Consequently, we conducted two sets of rheological property testing experiments. One set involved engineered sand concrete with a moderate dosage of admixtures, while the other set served as a control group with natural sand concrete. The results are shown in Figure 4, where (a) and (b) represent the variation of yield stress and relative plastic viscosity with time, respectively. Table 5 presents the calculated average values of yield stress and relative plastic viscosity for both mechanism sand concrete and natural river sand concrete.

In Figure 4a, it can be seen that starting from the observed point, the black lines are consistently positioned above the red lines. This indicates that the yield stress of the manufactured sand concrete is slightly lower than that of natural sand concrete. Additionally, it can be observed that the change in yield stress of artificial sand concrete with time is not significant. In Figure 4b, the trend of relative plastic viscosity of manufactured sand concrete and natural river sand concrete with time as a whole shows an increase and then decrease, but the increase and decrease in relative plastic viscosity of mechanism sand concrete is significantly earlier than that of natural sand concrete in time. From Table 5, it can be seen that the average yield stress and average plastic viscosity of mechanism sand concrete are lower than that of river sand concrete.

Based on the macroscopic observations of manufactured sand and natural river sand, as well as studies in the literature [42,43,44], manufactured sand has a larger axial diameter, a higher degree of plate-like and rod-like shapes, and rougher and more angular texture. This leads to a lower bulk density and a higher void ratio, increasing the probability of particle collisions during mortar shear motion. Furthermore, there is greater frictional force between particles in both the aggregate-to-aggregate and aggregate-to-matrix interfaces for manufactured sand, which increases the viscosity of the mixture and impairs its rheological properties, even as the yield stress and plastic viscosity rise. However, the experimental results for the rheological properties of artificial sand and natural river sand in this study contradict the above conclusion. This is because the addition of SP, PFA, and silica powder helps improve the particle grading of mechanism sand, increases the mortar content of artificial sand concrete, and compensates for the disadvantages of mechanism sand, such as its angular and rough surface texture. As a result, it reduces the frictional resistance between coarse aggregates, enhances flowability, serves as a physical water reducer, and lubricates the particles, thus improving the workability of manufactured sand concrete. Consequently, the yield stress and relative plastic viscosity of manufactured sand concrete are slightly lower than that of natural river sand concrete.

### 3.2. Compressive Strengths

#### 3.2.1. Box–Behnken Experimental Design and Significance Tests

1. Box–Behnken experimental design

**Figure 5:**
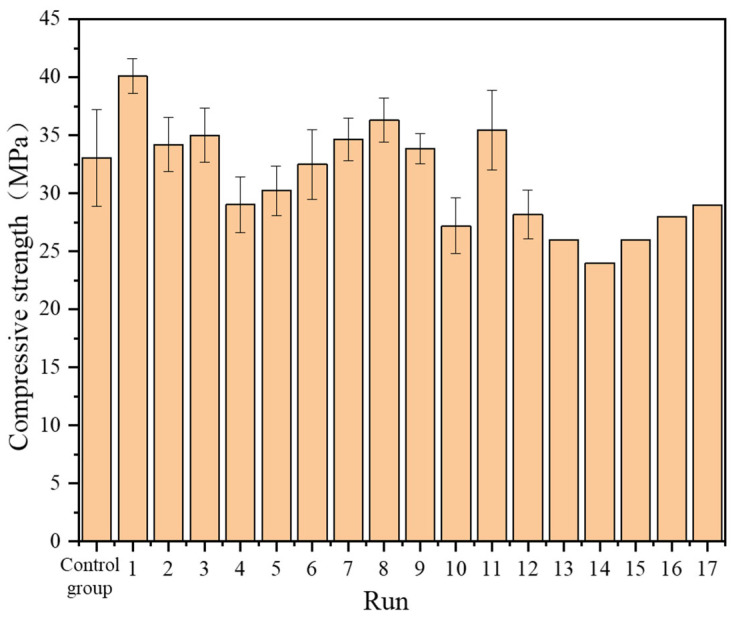
Results of Box–Behnken experiments.

Using uniaxial compressive strength as the response variable, the factors considered are SP content (X_1_), PFA content (X_2_), and SF content (X_3_). In the compressive strength test, samples with the same ratio shall be tested at least three times, and the compressive strength results shall be taken as the average of the three tests and rounded to an integer. In the compressive strength tests, results for Groups 1–12 and the control group represent the mean of three strength values, rounded to the nearest integer. For experiments in Groups 13–17, the Box–Behnken experimental design accounted for experimental errors by conducting five identical tests, hence representing one trial’s result. The Box–Behnken experimental factors and levels and the experimental results are presented in Table 6 and Table 7 and Figure 5.

Except for the control group, the least squares method is used to fit the experimental data of manufactured sand, excluding the control group, and the regression model is established as follows:

Y = 26.60 − 1.00X_1_ − 3.00X_2_ + 1.25X_3_ − 0.50X_1_X_3_ + 5.20X_1_X_1_ + 2.70X_2_X_2_ + 1.70X_3_X_3_ R^2^ = 0.83 (1)

2. Significance Tests

A variance analysis is performed on the established standard quadratic regression equation (Equation (1)), and the results are shown in Table 8. A significance test is conducted on the model through variance analysis, with a significance level set at 0.05. When *p* < 0.05, the indicator is considered significant; when *p* > 0.05, the indicator is considered not significant. As can be seen from Table 8, the quadratic regression model of compressive strength yields *p* < 0.05, and the multiple correlation coefficient R^2^ is 0.83, which shows that the regression equation can approximate the real surface well and the model can accurately predict the compressive strength of concrete.

In Table 8, the variance values (squares) for SP, PFA, and SF under single factors are 8.00, 72.00, and 12.50, respectively. Therefore, the significance levels of single factors on compressive strength are as follows: PFA has the greatest impact on the compressive strength of artificial sand concrete, followed by SF, and SP has the least impact. Similarly, as shown in Table 8, for the compounding effect, the compressive strength of artificial sand concrete is most significantly affected by SP and SF, and the interaction between SP and PFA, and PFA and SF have the same effect on compressive strength.

**Figure 6:**
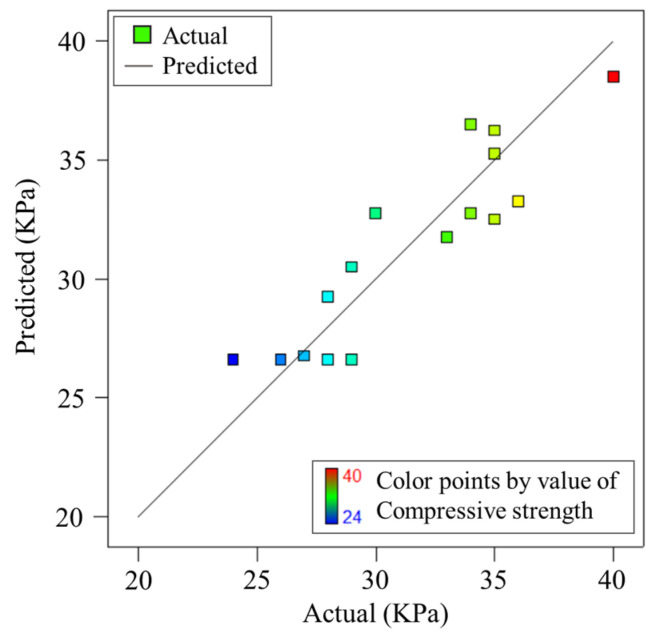
Response surface predicted and experimental values of compressive strength.

Figure 6 compares the predicted values and experimental values of compressive strength. It can be seen from the figure that the predicted values closely match the experimental values.

#### 3.2.2. Response Surface and Contour Analysis

Based on the mechanism sand concrete compressive strength regression equation, the effect of two interactions of SP, PEA, and SF on compressive strength is analyzed using response surface and contour analysis. One additional factor is controlled to be at an intermediate level when discussing the pattern of the two-by-two interaction effects on compressive strength. When discussing the effects of two-factor interactions on compressive strength, the other factor is kept at the intermediate level.

According to the previous section, the intermediate levels of SP, PEA, and SF dosage in this study are 10%, 15%, and 5% of the cement weight.

1. Compound doping of SP and PFA

**Figure 7:**
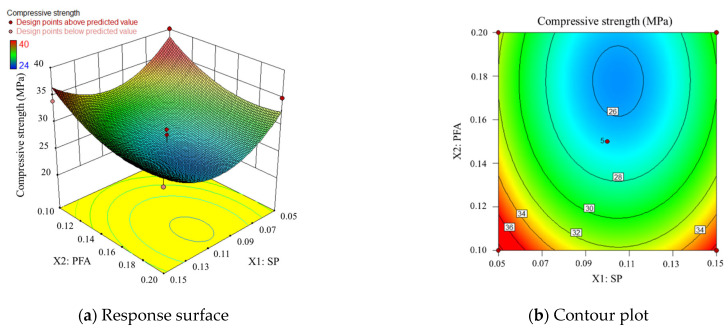
Response surface and contour plot corresponding to SP and PFA.

Figure 7 shows the response surface and contour lines for the joint action of SP and PFA, with SF held at the intermediate level. In Figure 7a, it can be seen that when SF is constant, the entire response surface forms a “reversed arch” shape with a minimum value. This suggests that with increasing SP and PFA content, the compressive strength initially decreases and then increases, with the maximum value occurring at the lowest levels of SP and PFA content within the experimental range provided in this study. Figure 7b contour lines reveal that the contour distribution in the lower-left corner of the image is denser, indicating that changes in the content of both SP and PFA have a larger impact on compressive strength fluctuations when their content is relatively low, and their impact trends are essentially the same. It is worth noting that the term “relatively low content” here corresponds to the range set in this study. Taking PFA as an example, the content range in this study is 10% to 20% of cement weight. Therefore, “relatively low content” in this context means content close to 10%, but not less than 10%. The subsequent discussions also follow the same rules.

2. Compound doping of SP and SF

**Figure 8:**
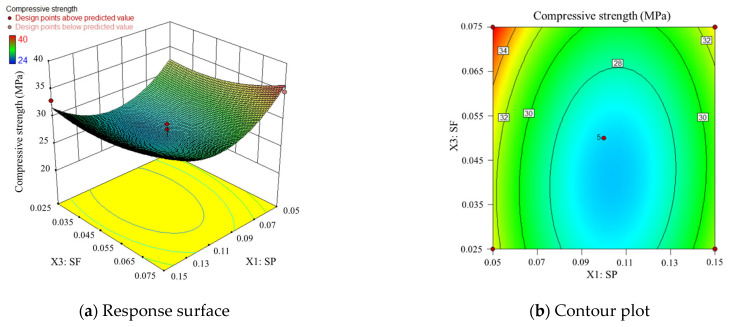
Response surface and contour plot corresponding to SP and SF.

Similarly, the PFA is at a moderate level, and the response surface and contours for SP and SF are shown in Figure 8. In Figure 8a, compared to Figure 7a, both three-dimensional surface plots exhibit an “inverted arch” shape. Unlike Figure 7a, the contour lines in Figure 8a are sparser at lower compressive strengths. This suggests that when the PFA content is held constant, variations in the levels of SP and SF within a moderate range have a relatively small impact on compressive strength, with SP having a greater influence than SF. The density of contour lines in Figure 8b indicates that changes in the SP content have a greater fluctuating effect on compressive strength when SP content is low and SF content is high, with the influence of SP content changes being greater than those of SF within the specified range of this study. This implies that when PFA content is 15% of the cement weight and SF content is less than 4.5%, changes in SP content result in smaller fluctuations in compressive strength compared to the case where SF content is greater than 4.5%. Additionally, when SP content varies within the range of 7% to 13%, changes in SF content also result in smaller fluctuations in compressive strength compared to changes in SP content (within the experimental range provided in this study) at levels below 7% and above 13%.

3. Compound doping of PFA and SF

**Figure 9:**
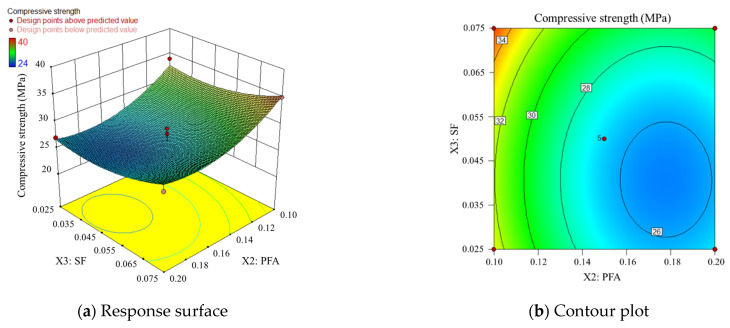
Response surface and contour plot corresponding to PFA and SF.

The corresponding response surfaces and contours for PFA and SF are shown in Figure 9, at which point the SP is at a moderate level. Figure 9 indicates that the contour lines are denser when PFA content is lower, suggesting that when PFA content is higher, variations in SF content have a relatively small fluctuating effect on compressive strength, whereas when PFA content is lower, the effect on compressive strength is more significant. Figure 9a shows that there is still a minimum value on the entire response surface when PFA content is higher and SF content is lower. Combining Figure 7, Figure 8 and Figure 9, it can be observed that in this study, the effect of SP content on overall compressive strength is more significant than that of PFA and SF. Moreover, when SP and PFA content are at their minimum levels and SF content is at its maximum, minimum SP and PFA admixture and maximum SF admixture are found for relatively maximum compressive strength of manufactured sand concrete, but the present study failed to arrive at the optimum admixture of SP, PFA, SF.

## 4. Conclusions

In this study, RSM was used to study the compressive strength of manufactured sand concrete compounded with SP, PFA, and SF. Additionally, research was conducted on the profile of the sand and the rheological properties of concrete, leading to the following conclusions.

1. The combined use of SP, PFA, and SF improved the particle size distribution of artificial sand, mitigating its angularity and surface roughness shortcomings. This combination acts as a physical water reducer and enhances interparticle lubrication, thereby improving the rheological properties of artificial sand concrete.
2. RSM considers random experimental errors, predicting the experimental results within variable ranges. The predictive model obtained from experimental results through RSM is continuous. This study effectively predicts the compressive strength of artificial sand concrete under different admixture levels and provides continuous analysis of various levels of the experiment. The study utilized multiple regression analysis to establish a predictive model for the compressive strength of siliceous artificial sand concrete within the ranges of SP, PFA, and SF, whose contents are 5–15%, 10–20%, and 2.5–7.5% respectively. The multiple correlation coefficient (R2) of the predictive model was 0.83, indicating its high reliability.
3. SP had the greatest effect on the compressive strength of mechanism sand concrete and SF had the least effect. For compounding factors, SP and PFA had the most significant effect on the compressive strength of artificial sand concrete, and PFA and SF had the least effect.
4. Response surface and contour analyses are performed at SP, PFA, and SF dosages of 10%, 15%, and 5% of the cement weight. With increasing SP, PFA, and SF content, the compressive strength shows a decrease and then an increase. The relative minimum compressive strength is observed when SP content is moderate, SF content is low, and PFA content is high. When SP and PFA content are at their minimum levels and SF content is at its maximum, the compressive strength is relatively highest. However, this study does not determine the optimal content levels for SP, PFA, and SF.

## Figures and Tables

**Table 1:** Composition of cement.

Constituents	SiO_2_	Al_2_O_3_	Fe_2_O_3_	CaO	MgO	SO_3_
Content (wt%)	20.81	4.54	3.15	64.22	2	2.5

**Table 2:** Composition of PFA.

Constituents	SiO_2_	Al_2_O_3_	Fe_2_O_3_	CaO	TiO_2_	K_2_O	MgO
Content (wt%)	32.61	24.54	3.45	4.42	0.93	0.84	0.56

**Table 3:** Composition of SF.

Constituents	SiO_2_	Al_2_O_3_	Fe_2_O_3_	CaO	SO_3_	K_2_O	MgO	Na_2_O
Content (wt%)	92.5	0.2	0.6	0.12	0.44	1.52	0.15	0.37

**Table 4:** Composition table of concrete (expressed as a percentage by weight of cement).

Run	Cement	Crushed Stone	Sand	Water	X_1_	X_2_	X_3_
Control group	100%	150%	2.25%	50%	0	0	0
1					5%	10%	5%
2					15%	10%	5%
3					5%	20%	5%
4					15%	20%	5%
5					5%	15%	2.5%
6					15%	15%	2.5%
7					5%	15%	7.5%
8					15%	15%	7.5%
9					10%	10%	2.5%
10					10%	20%	2.5%
11					10%	10%	7.5%
12					10%	20%	7.5%
13					10%	15%	5%
14					10%	15%	5%
15					10%	15%	5%
16					10%	15%	5%
17					10%	15%	5%

**Table 5:** Comparison of yield stress and relative plastic viscosity.

Average Value	Yield Stress (Nm)	Plastic Viscosity (Ns)
Natural sand	1.23	7.49
Manufactured sand	1.06	7.31

**Table 6:** Factors and levels of Box–Behnken experiments.

Factors	−1	0	1
X_1_ (SP content)	0.05	0.1	0.15
X_2_ (PFA content)	0.1	0.15	0.2
X_3_ (SF content)	0.025	0.05	0.075

**Table 7:** Results of Box–Behnken experiments.

Run	X_1_	X_2_	X_3_	Y/Compressive Strength (MPa)
Control group	0	0	0	33
1	5%	10%	5%	40
2	15%	10%	5%	34
3	5%	20%	5%	35
4	15%	20%	5%	29
5	5%	15%	2.5%	30
6	15%	15%	2.5%	33
7	5%	15%	7.5%	35
8	15%	15%	7.5%	36
9	10%	10%	2.5%	34
10	10%	20%	2.5%	27
11	10%	10%	7.5%	35
12	10%	20%	7.5%	28
13	10%	15%	5%	26
14	10%	15%	5%	24
15	10%	15%	5%	26
16	10%	15%	5%	28
17	10%	15%	5%	29

**Table 8:** Variance analysis of response surface experimental results.

Source	Squares	df	Square	Value	Prob > F	
Model	264.06	9	29.34	3.82	0.0454	significant
X1-SP	8.00	1	8.00	1.04	0.3412	
X2-PFA	72.00	1	72.00	9.39	0.0182	
X3-SF	12.50	1	12.50	1.63	0.2425	
X1 X2	5.684 × 10^−14^	1	5.684 × 10^−14^	7.410 × 10^−15^	1.0000	
X1 X3	1.00	1	1.00	0.13	0.7287	
X2 X3	5.684 × 10^−14^	1	5.684 × 10^−14^	7.410 × 10^−15^	1.0000	
X1 X1	113.85	1	113.85	14.84	0.0063	
X2 X2	30.69	1	30.69	4.00	0.0856	
X3 X3	12.17	1	12.17	1.59	0.2482	
Residual	53.70	7	7.67			
Lack of Fit	38.50	3	12.83	3.38	0.1352	not significant
Pure Error	15.20	4	3.80			
Cor Total	317.76	16				

## Data Availability

Data are contained within the article.

## Abbreviations

RSM	response surface method
SP	stone powder
PFA	pulverized fuel ash
SF	silicon fume

## References

[1] Sundaralingam K., Peiris A., Sathiparan N. Manufactured Sand as River Sand Replacement for Masonry Binding Mortar. Proceedings of the Moratuwa Engineering Research Conference (MERCon 2021)/7th International Multidisciplinary Engineering Research Conference.

[2] Ferrández D., Alvarez M., Saiz P., Zaragoza-Benzal A. (2022). Recovery of mineral wool waste and recycled aggregates for use in the manufacturing processes of masonry mortars. Processes.

[3] Tamanna N., Tuladhar R., Sivakugan N. (2020). Performance of recycled waste glass sand as partial replacement of sand in concrete. Constr. Build. Mater..

[4] Chen L., Ma H., Sun Z., Zhang Y. (2022). Flowability of multi-sized mixed particles based on shotcrete aggregates. Powder Technology..

[5] Liu Y., Guo Y., Li H., Wang H. (2020). Experimental study on the effect of recycled aggregate from construction waste on the transportation performance of mine filling paste. Shandong Univ. Sci. Technol. Nat. Sci..

[6] Yin Y. (2017). Research on the Performance and Mix Ratio of Mechanized Sand Concrete.

[7] Ma H., Sun Z., Ma G. (2022). Research on compressive strength of manufactured sand concrete based on response surface methodology (RSM). Appl. Sci..

[8] Chen L., Sun Z., Li P., Ma H., Pan G. (2022). DEM simulation of the transport of mine concrete by a screw feeder. J. Braz. Soc. Mech. Sci. Eng..

[9] Arulmoly B., Konthesingha C., Nanayakkara A. (2021). Performance evaluation of cement mortar produced with manufactured sand and offshore sand as alternatives for river sand. Constr. Build. Mater..

[10] Murat O., Murat G. (2014). Assessment of optimum threshold and particle shape parameter for the image analysis of aggregate size distribution of concrete sections. Opt. Lasers Eng..

[11] Mundra S., Sindhi P.R., Chandwani V., Nagar R., Agrawal V. (2016). Crushed rock sand–An economical and ecological alternative to natural sand to optimize concrete mix. Perspect. Sci..

[12] Zhang Q., Zou J., Chi M., Jiao Y., Yan X. (2023). On the strong mining-induced earthquakes induced by the fracturing of key strata during deep coal mining. Int. J. Geomech..

[13] Wang H., Gao M., Gao Y., Chen Q. (2021). Experimental study on dynamic characteristics of calcareous sand solidified by polymer. Shandong Univ. Sci. Technol. Nat. Sci..

[14] Martins P., Diane G., Robert L. (2016). An Investigation into the Use of Manufactured Sand as a 100% Replacement for Fine Aggregate in Concrete. Materials.

[15] Wang J., Xue C., Zhang Y., Li Q., Han Y., Qiao H. (2023). Study of Early-Age Hydration, Mechanical Properties Development, and Microstructure Evolution of Manufactured Sand Concrete Mixed with Granite Stone Powder. Materials.

[16] Li T., Tier L. (2022). Microscopic mechanism analysis of the influence of stone powder with different replacement ratio on concrete performance. Arab. J. Geosci..

[17] Meera M., Anuj Supravin K., Gupta S. (2020). Importance of moisture correction in fine powder materials for concrete. Mater. Today Proc..

[18] Bentz D.P., Ardani A., Barrett T., Jones S.Z., Lootens D., Peltz M.A., Sato T., Stutzman P.E., Tanesi J., Weiss W.J. (2015). Multi-scale investigation of the performance of limestone in concrete. Constr. Build. Mater..

[19] Voglis N., Kakali G., Chaniotakis E., Tsivlis S. (2005). Portland-limestone cements. Their properties and hydration compared to those of other composite cements. Cem. Concr. Compos..

[20] Otman M.M.E., Sghaiar N., Mohammed S., Gamil M.S.A., Abdullah M.Z. (2022). Performance of self-compacting concrete incorporating wastepaper sludge ash and pulverized fuel ash as partial substitutes. Case Stud. Constr. Mater..

[21] Kim D.G., Lee S.T. (2021). Mechanical Properties of Ternary Blended Cement Concrete Incorporating Pulverized Reject Ash. Collect. Pap. Korean Road Soc..

[22] Ding X., Li C., Xu Y., Li F., Zhao S. (2016). Experimental study on long-term compressive strength of concrete with manufactured sand. Constr. Build. Mater..

[23] Wang Q., Jiang M., Xu G., Zhao J., Zhao B. (2018). The Effect of Machine-made Sand and Stone Powder Content on the Strength of Pumped Concrete. Concrete.

[24] Knop Y., Peled A., Cohen R. (2014). Influences of limestone particle size distributions and contents on blended cement properties. Constr. Build. Mater..

[25] Li P., Brouwers H.J.H., Chen W., Yu Q. (2020). Optimization and characterization of high-volume limestone powder in sustainable ultra-high performance concrete. Constr. Build. Mater..

[26] Ding H., Song X., Han J. (2016). The Affection to the Performance of Concrete Cracking of Fly Ash and Slag. Fly Ash Compr. Util..

[27] Peng Y., Xiao J., Gao D., Tang Y., Chen S., Yang Z. (2018). The effect of stone powder content in machine-made sand on the performance of cement mortar. Concrete.

[28] Fu T., Liang J., Lan Y., Xiao Y., Wei J. (2022). Effect of Stone Powder Content on Mechanical Properties of Manufactured Sand Concrete. Iran. J. Sci. Technol. Trans. Civ. Eng..

[29] Box G.E.P., Behnken D.W. (1960). Some new three level designs for the study of quantitative variables. Technometrics.

[30] Kumar R. (2020). Modified mix design and statistical modelling of lightweight concrete with high volume micro fines waste additive via the Box-Behnken design approach. Cem. Concr. Compos..

[31] Khudhair M.H., Al-Anweh A.M., Nomaan M.H., Berradi M., Hsissou R., Bekhta A., Elyoubi M.S., Elharfi A. (2019). Response surface modeling of compressive strength of high performance concrete formulated by a high water reducing and setting accelerating superplasticizer. Box-Behnken experimental design. Chem. Technol. Metall..

[32] (2020). Common Portland Cement.

[33] (2022). Standard Specification for Portland Cement.

[34] (2018). Standards of Water for Concrete.

[35] (2018). Technical Code for Engineering of Ground Anchorages and Shotcrete Support.

[36] Nordin N., Abdullah M.M.A.B., Fakri W.M.N.R.W., Tahir M.F.M., Sandu A.V., Hussin K., Zailani W.W.A. (2019). Exploration on Fly Ash Waste as Global Construction Materials for Dynamics Marketability. Int. Conf. Green Des. Manuf..

[37] Khan M.I., Siddique R. (2011). Utilization of silica fume in concrete: Review of durability properties. Resour. Conserv. Recycl..

[38] Hamada H.M., Abed F., Katman H.Y.B., Humada A.M., Al Jawahery M.S., Majdi A., Yousif S.T., Thomas B.S. (2023). Effect of silica fume on the properties of sustainable cement concrete. J. Mater. Res. Technol..

[39] Kansal C.M., Goyal R. (2021). Effect of nano silica, silica fume and steel slag on concrete properties. Mater. Today Proc..

[40] (2019). Standard for Test Methods of Concrete Physical and Mechanical Properties.

[41] Xue D. (2020). Determination of uniaxial compressive strength of intact rock. Shandong Univ. Sci. Technol. Nat. Sci..

[42] Altuki R., Ley M.T., Cook D., Jagan G.M., Praul M. (2022). Increasing sustainable aggregate usage in concrete by quantifying the shape and gradation of manufactured sand. Constr. Build. Mater..

[43] Barry M., Clément M., Rangeard D., Jacquet Y., Perrot A. (2023). Manufactured crushed sand: Packing fraction prediction and influence on mortar rheology. Mater. Struct..

[44] Skare E.L., Sheiati S., Cepuritis R., Mørtsell E., Smeplass S., Spangenberg J., Jacobsen S. (2022). Rheology modelling of cement paste with manufactured sand and silica fume: Comparing suspension models with artificial neural network predictions. Constr. Build. Mater..

