# Correction: Gao et al. Research on Compressive Strength of Manufactured Sand Concrete Based on Response Surface Methodology. *Materials* 2024, *17*, 195

**DOI:** 10.3390/ma17071640

**Published:** 2024-04-03

**Authors:** Kang Gao, Zhenjiao Sun, Hui Ma, Guanguo Ma

**Affiliations:** 1 College of Safety and Environmental Engineering, Shandong University of Science and Technology, Qingdao 266590, China; 2 School of Safety and Engineering, China University of Mining and Technology, Xuzhou 221116, China

In the original publication [1], there was a mistake in the photos inserted as published. Figure 2 mistakenly contained photos of concrete pieces from another experiment. The corrected Figure 2 appears below. 

The authors state that the scientific conclusions are unaffected. This correction was approved by the Academic Editor. The original publication has also been updated.

## Figures and Tables

**Figure 2:**
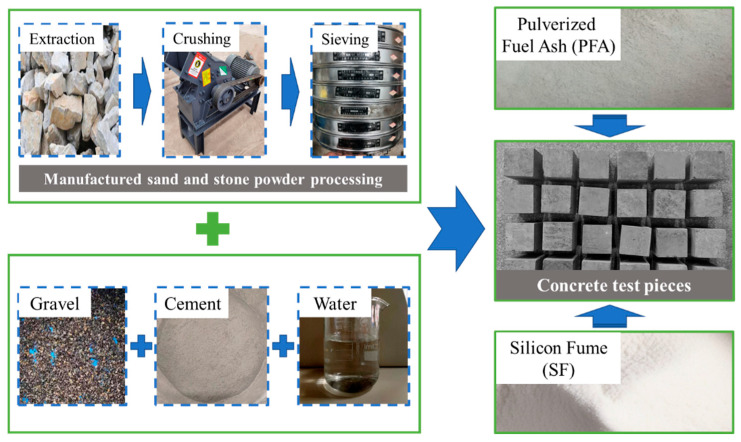
The fabrication process of the test pieces.
